# Burst Feeding of *Pelagia noctiluca* ephyrae on Atlantic Bluefin Tuna (*Thunnus thynnus*) Eggs

**DOI:** 10.1371/journal.pone.0074721

**Published:** 2013-09-19

**Authors:** Ana Gordoa, José Luis Acuña, Roser Farrés, Kathrin Bacher

**Affiliations:** 1 Department of Marine Ecology, Centro de Estudios Avanzados de Blanes, Spanish National Research Council (CSIC), Blanes, Gerona, Spain; 2 Departamento de Biología de Organismos y Sistemas, Universidad de Oviedo, Oviedo, Asturias, Spain; Institute of Marine Research, Norway

## Abstract

This study investigates the predation of *P* *. noctiluca* ephyrae on Atlantic Bluefin tuna (ABFT) eggs under different experimental conditions. The specific factors considered in the experimental design were: a) water mix conditions to explore predation under two-dimensional (2D) and three-dimensional (3D) prey distributions, b) prey density to investigate the ingestion rate capacity, and c) incubation time to inspect gut saturation. The eggs and jellyfish ephyrae were collected during the 2012 ABFT spawning survey off Ibiza (Balearic Isl., Western Mediterranean). The results showed that the proportion of feeding ephyrae increased with size. The mean clearance rate of feeding ephyrae, 4.14 L h^-1^, was the highest ever recorded for ephyrae. Under calm conditions the eggs floated at the surface (2D spatial arrangement) and the clearance rates, at low prey densities, were at least twice those under mixed conditions (3D spatial arrangement). At high prey density, clearance rate did not differ between mix conditions, probably due to the fast gut saturation, which was reached in c.a. 15 min, as revealed by time series observations of gut contents. The fast saturation of ephyrae and their slow digestion time of approximately 18 h suggest the existence of a diel feeding periodicity. We conclude that in the Western Mediterranean, *P* *. noctiluca* ephyrae are capable of predating on ABFT eggs, a highly pulsed and spatially restricted resource that potentially switches from a 3D to a 2D configuration in the absence of wind-generated turbulence. The *P* *. noctiluca* and Atlantic Bluefin tuna egg system might represent an example of a general mechanism linking pelagic and neustonic food webs.

## Introduction

The Atlantic bluefin tuna (hereafter *Thunnus thynnus*, ABFT) is a highly migratory species targeted on both sides of the Atlantic by different fisheries due to its extraordinary commercial value. This species is composed of two stocks which share Atlantic feeding grounds and migrate to spawn in warmer seas. The Western stock spawns in the Gulf of Mexico [1] and the eastern stock in the Mediterranean [2]. Tuna fishing commenced around 7000 BC in the Mediterranean [3]. Since the 16^th^ century, the early fishing modalities have been replaced gradually by traps [4], and in the late 1990s the increase in market demand and the incorporation of capture-based aquaculture [5] led to a high risk of overfishing. However, the ABFT is certainly exposed to other hazards, since its biogeographic range contracted decades before the rise in fishing intensity. This contraction had been reported since the 1950s [6,7], but the reasons for its disappearance in certain areas are still unclear [8]. In view of the weak condition of the stocks at the beginning of this century, the International Commission for the Conservation of Atlantic Tunas (ICCAT) established a multiannual recovery plan in 2007. However, the rebuilding capacity of fish populations also depends on fishing-independent factors, in particular those responsible for recruitment success or offspring survival during the early life stages of development. Mortality during the egg and larval stages is thought to play a major role in determining survival and latter recruitment into adult stocks [9,10]. There are a wide variety of factors inducing fish egg mortality [11-13], although predation is generally considered the main cause [14-16].

Jellyfishes are top planktonic predators [17], generally considered detrimental to fish populations through competition for zooplankton prey [18] or directly by predation on fish eggs and larvae [19]. Jellyfish predation on fish eggs and larvae has been confirmed from results of experimental studies [20-23]. In addition, a positive selection for ichthyoplankton has been reported [24-27]. In the Mediterranean, *P* *. noctiluca* , a warm-water holoplanktonic scyphomedusae [28], is abundant and widespread in various regions [29-33], showing large fluctuations with massive outbreaks correlated with warm, dry conditions [34]. This species represents the most abundant and most venomous jellyfish in the Mediterranean [35]. *P* *. noctiluca* is an opportunistic predator [31]; therefore, its impact on fish populations may vary with regions and will depend on its spatial and temporal overlap with the early life stages of fish populations.

The impact of *P* *. noctiluca* on ABFT spawning is unknown. However, increasing evidence of their spatio-temporal overlap prompts an evaluation of this predation potential. The abundance of this jellyfish in Balearic waters is increasing and its swarms enter the Mediterranean following the progression of Atlantic surface waters [36]. This is the same migratory displacement process and route known for ABFT spawning [4,37,38]. Balearic waters represent the most important ABFT spawning area in the Mediterranean [39,40], where spawning seems to takes place mostly in offshore mixed waters of the frontal areas in the confluence of Atlantic and Mediterranean water masses [41]. There is evidence from different studies that higher densities of this jellyfish species also occur in the vicinity of frontal areas [27,30]. The results from field studies on spawning behavior, aboard ABFT transport cages in Balearic waters, showed a clear nocturnal concurrence of ABFT spawning and *P* *. noctiluca* larvae at the sea surface [42], where jellyfish larvae appeared to exhibit predatory behavior on ABFT eggs.

Solid experimental evidence of predation of *P* *. noctiluca* ephyrae on ABFT eggs must be secured before attempting any speculation on the potential impact of this jellyfish on ABFT populations. The diurnal scarcity and nocturnal abundance of *P* *. noctiluca* at the sea surface in the ABFT spawning region [42] is indicative of the well-known pattern of diel vertical migration of this species [30,35,43,44], enabling foraging throughout a wide range of water column depths. In contrast, freshly laid ABFT eggs are positively buoyant and rapidly accumulate on a single layer at the surface of experimental beakers (Gordoa, unpublished observation). *P* *. noctiluca* captures prey while cruising [30], a feeding mechanism which seems hardly compatible with a two-dimensional prey distribution [45].

ABFT transport cages transiting Balearic waters are used to monitor ABFT spawning patterns and serve as a unique opportunity to concurrently obtain live specimens of ABFT eggs and *P* *. noctiluca* for an experimental test of predation. In June 2012, we used this opportunity to conduct predation experiments in a portable laboratory in a coastal aquarium-cave in the island of Ibiza (W Mediterranean). In this study we investigated the predation of *P* *. noctiluca* ephyrae on ABFT eggs under different experimental conditions. We explored the role of positive egg buoyancy as a strategy to evade ephyrae predation by measuring predation rates in mixed experimental beakers, where the eggs had a 3D distribution, and still experimental beakers, where eggs accumulated at the surface (2D). We also tested the effect of prey density and incubation time on predation rates and digestion time. Video observations were recorded to provide supplementary information on predatory capture conducts. Our results provide experimental evidence of predation of *P* *. noctiluca* ephyrae on ABFT eggs, using highly efficient alternative predation mechanisms for 3D (water column) and 2D (neuston) prey fields.

## Methods

### Experiment logistics

**Figure 1:**
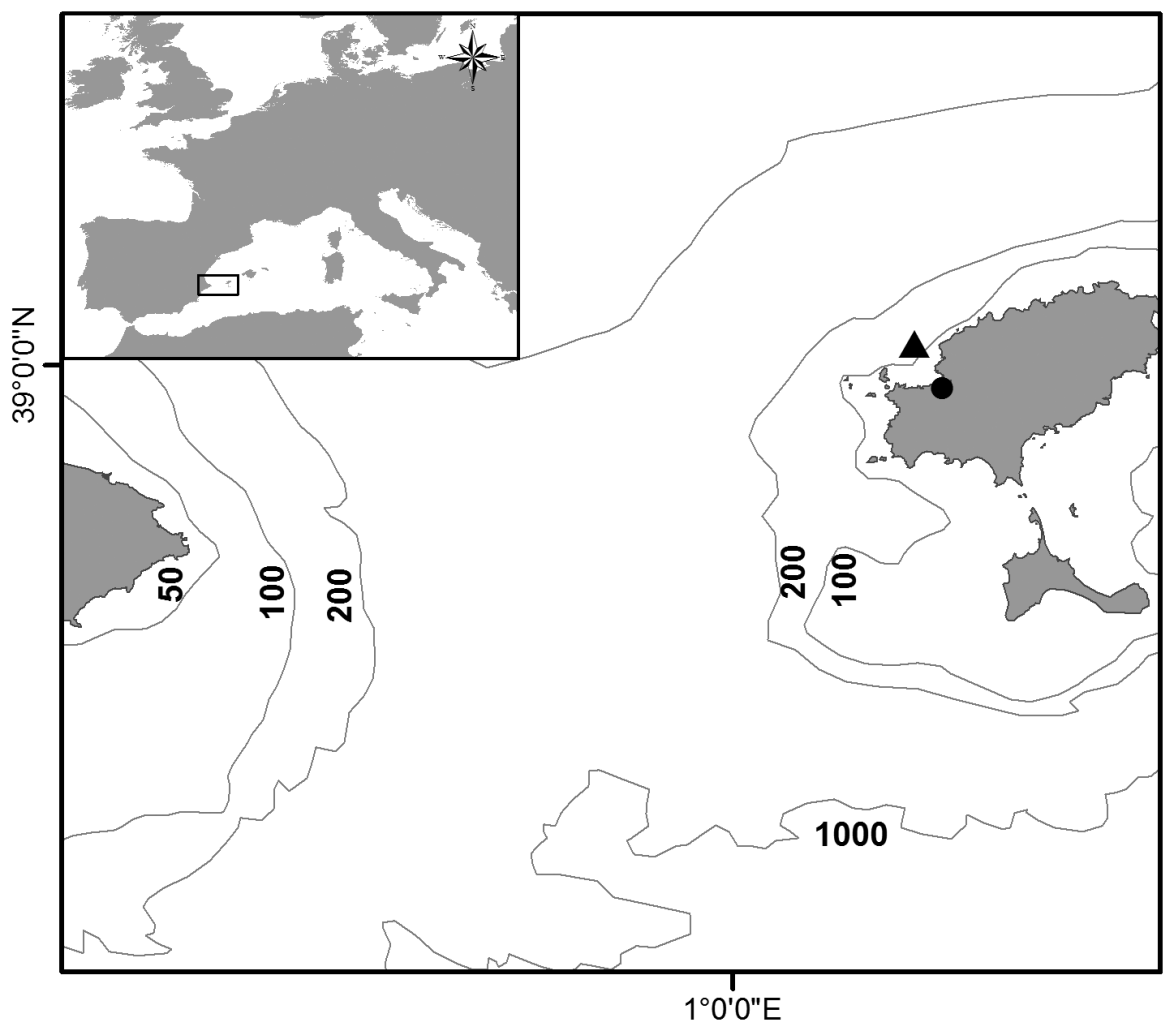
Map showing the location of the field laboratory (●) and of the tuna transport cage (▲).

Experiments were conducted at the Cap Blanc Aquarium, a coastal cave located in Ibiza (Figure 1). The permission to perform the study on this site was given by the town council of Sant Antoni de Portmany (Ibiza) and the Aquarium Cap Blanc, a private company that holds the concession of the aquarium. This location was chosen for its proximity to the ABFT spawning grounds, in order to minimize transport time of jellyfish and ABFT eggs. The samples were collected during the 2012 spawning survey using a bongo frame fitted with 0.3 mm mesh nets and towed from the rear of an ABFT transport cage at a fixed depth of 3 m [42]. The cage was moved close to the aquarium during 13^th^ to 27^th^ June to facilitate transport of specimens to the field laboratory.

Sampling was carried out at night during the ABFT spawning window (2: 00 to 5: 00 a.m. local time). Live fish eggs and jellyfish were carefully transferred to transport containers filled with 0.3 mm filtered water for transport to the field laboratory.

### Experiment layout

Prior to the experiments, the eggs were kept in two 40-L aerated aquaria and the jellyfish in two 6-L aquaria without aeration. All the aquaria were filled with 3 mm filtered seawater adjusted to the seawater temperature at the time of sampling (23 ± 0.3 °C). Every two days, the aquaria were cleaned and filled with fresh eggs. Due to space limitations, replicated experimental treatments were not done simultaneously but sequentially one at a time, between 10:00 and 18:00, with replicates adequately interspersed in time.

The study considered four treatments arranged in a 2-way orthogonal design with a prey density factor with high (12 eggs L^-1^) and low (6 eggs L^1^) density levels and a mixing regime factor with two levels, stirred and still experimental suspension (these treatments led to a homogeneous, 3D distribution of the eggs within the experimental beaker and to a 2D surface accumulation, respectively) (Table 1). The low egg concentration was selected according to other similar experiments with gelatinous predators [14,46] to ensure being in the linear segment of the functional response curve, and doubling the prey density to enable a differential concentration response. Gentle mixing conditions were achieved by using a methacrylate comb with 5 teeth, each 1.5 cm wide and 1.5 cm apart and penetrating 2 cm into the water. The comb was connected to an electric motor rotating at 10 rpm and changing direction every 2 seconds.

**Table 1:** List of treatments used in the experiments.

			Incubation time			
Mixing regime	Egg concentration	5’	10’	15’	25’	120’
unstirred	low (6 egg L^-1^)	2(u)	2(u)	4(s)	3(s)	4(s)
unstirred	high (12 egg L^-1^)			3(s)		
stirred	low (6 egg L^-1^)			4(u)		
stirred	high (12 egg L^-1^)			4(s)		

Number of replicates for each treatment and whether the jellyfish guts were under saturated (s) or unsaturated (u) conditions with Atlantic Bluefin Tuna eggs, according to our results.

We used 10 jellyfish per incubation, which lasted 15 minutes. The scarcity of *P* *. noctiluca* in 2012 imposed limitations on the availability of ephyrae of different sizes for experimentation. Although the size range of collected jellyfish was 3-13 mm, most of them were smaller than 7 mm (median = 5 mm): consequently, bigger individuals were under-represented in the experiments. A known number of eggs were added to 6-L experimental beakers after being counted on a Petri disc. The eggs were gently spread over the water surface, and under still experimental conditions the ephyrae were introduced immediately afterwards, while under stirred conditions they were introduced once the eggs were fully mixed in the beaker. At the end of each experiment each jellyfish was removed from the tanks and placed alive under a stereo-microscope for measuring and counting the numbers of eggs inside its gut.

Also interspersed with the treatments of the previous experiment, we examined the effect of incubation time at a prey concentration of 6 eggs L^1^ without stirring (Table 1). For this, we conducted pairs of replicate incubations lasting 5, 10, 25 and 120 minutes, plus the four 15-minute replicates of the previous experiment (Table 1). Randomly and from different treatments, some of the jellyfish which had eggs in their guts after the incubation were kept in sealed 50-mL bottles and examined every 5 hours to monitor egg digestion and/or regurgitation, allowing a crude estimation of digestion time.

### Calculation of feeding rates

For each of the experimental beakers, individual ingestion rates were calculated as

$$IR=\frac{e}{tN}$$

where *e* is the sum of eggs counted inside the guts of all larvae in the incubation, *t* is the incubation time and *N* is the total number of ephyrae which were feeding during the incubation.

Population clearance rates were calculated following Moller et al. (2010) [47] as

$$CR=\frac{V\left[\ln\left(C_{i}\right)-\ln\left(C_{f}\right)\right]}{tN}$$

where *V* is the volume of water in the incubation container (L) and *C*_*i*_ and *C*_*f*_ are the initial and final egg concentrations (eggs L^-1^) in the incubation flask. *C*_*i*_ was calculated as the initial concentration of eggs in the incubation flask, while *C*_*f*_ was estimated by subtracting the number of eggs observed in the guts of the ephyrae from the initial number of eggs in the container and dividing by *V*.

### Statistical analysis

The response of the proportion of feeding ephyrae (p), the individual ingestion rate (IR) and the individual clearance rate (CR) to mixing conditions and egg concentrations was analyzed with a two-way ANCOVA with mean ephyra size as covariate. Variables were log-transformed to account for allometric power relationships between the feeding rates and body length, and were checked for normality, homogeneity of variances and homogeneity of slopes prior to analysis. In the case of IR, there was a significant difference among regression slopes between treatments (P=0.04). However, the regression lines showed good fits and appeared nearly parallel on visual inspection, so we followed Quinn & Keough [48] and applied ANCOVA under an assumption of homogeneity of slopes. In addition, there were differences in the range of ephyrae size between treatments, so our ANCOVA adjustment for the effect of size involves some degree of extrapolation outside the ephyra size range for each treatment. Finally, one replicate was lost, which led to an unbalanced design, dealt with by using a type III sum of squares.

## Results

**Figure 2:**
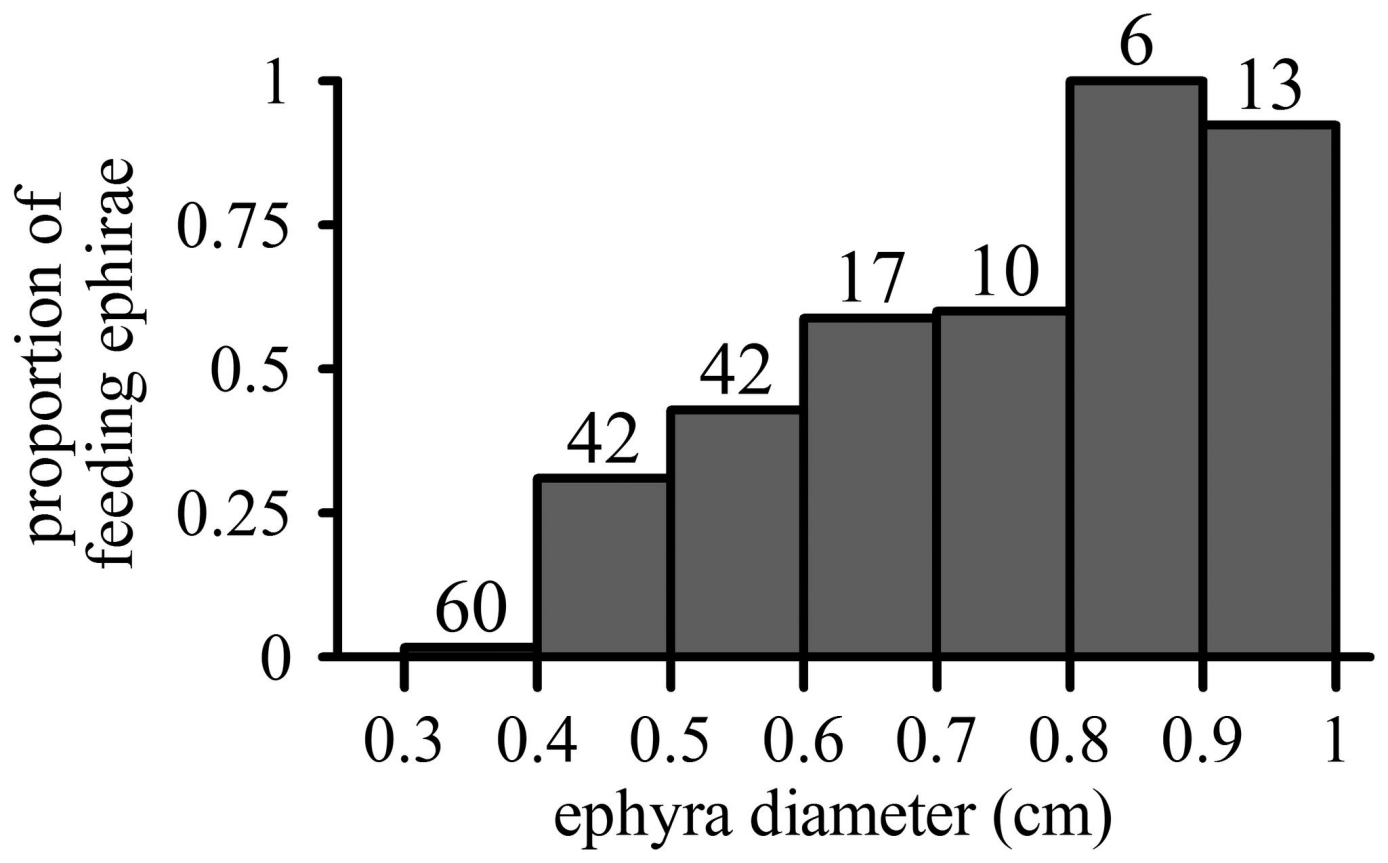
Proportion of feeding ephyrae in all of the experimental beakers for each of 7 different size classes. The first and the last classes are open. The numbers on top of each bar indicate the number of individuals.

**Figure 3:**
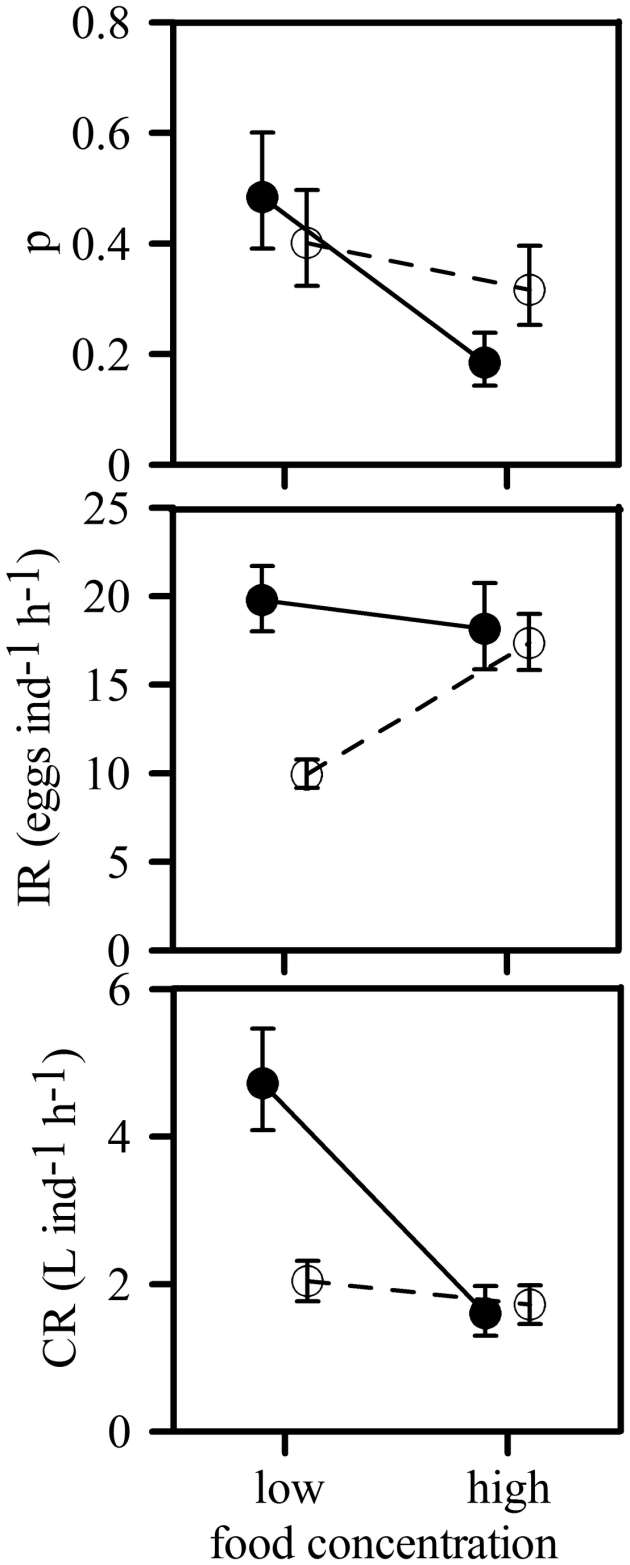
Effect of egg concentration and mixing regime on the proportion of feeding ephyrae (p), egg ingestion rate (IR) and egg clearance rate (CR) of *P* *. noctiluca* ephyrae feeding on Atlantic Bluefin tuna eggs. Dots represent geometric means adjusted to the mean ephyra size, which was 0.50 mm for p (includes all ephyrae used in each incubation) and 0.66 mm for IR and CR (includes only feeding ephyrae). Deviations correspond to ±10^SE^, where SE is the standard error of the log-transformed variables. Filled and empty dots correspond to the unstirred and stirred treatments, respectively.

After the experimental incubations, not all the jellyfish had eggs inside their gastric pouches, indicating that not all were feeding actively on ABFT eggs. The proportion of feeding ephyrae increased with size, with nearly all individuals larger than 8 mm containing ABFT eggs in their guts (Figure 2). In the experiments, the number of feeding ephyrae varied between 0 and 7 out of the ten animals used per incubation. The proportion of feeding ephyrae depended significantly on mean ephyrae size (P=0.030) and on egg concentrations (P=0.020), but was unaffected by mixing conditions (P>0.05, Table 2, Figure 3).

**Table 2:** ANCOVA table for the ephyrae response to different treatments.

**Source**		**df**	**MS**	**F**	**P**
*Dependent variable: p*					
egg density		1	0.25	7.63	0.020*
mixing		1	0.02	0.37	0.444
egg density x mixing		1	0.07	2.03	0.185
logsize (all ephyrae)		1	0.21	6.42	0.030*
*Dependent variable: IR*					
egg density		1	0.03	15.38	0.040*
mixing		1	0.07	28.67	0.004*
egg density x mixing		1	0.03	6.83	0.026*
logsize (feeding ephyrae)		1	0.39	79.70	0.000*
*Dependent variable: CR*					
egg density		1	0.19	16.27	0.002*
mixing		1	0.08	6.39	0.030*
egg density x mixing		1	0.07	5.66	0.039*
logsize (feeding ephyrae)		1	0.54	45.20	0.000*

Dependent variables were the proportion of feeding ephyrae (p , the individual ingestion rate (IR) and the individual clearance rate (CR). It is a two-way, fully orthogonal design with two fixed factors, mixing regime and food concentration, and mean ephyra size as covariate. Asterisk indicates significant (<0.05) P-values.

**Figure 4:**
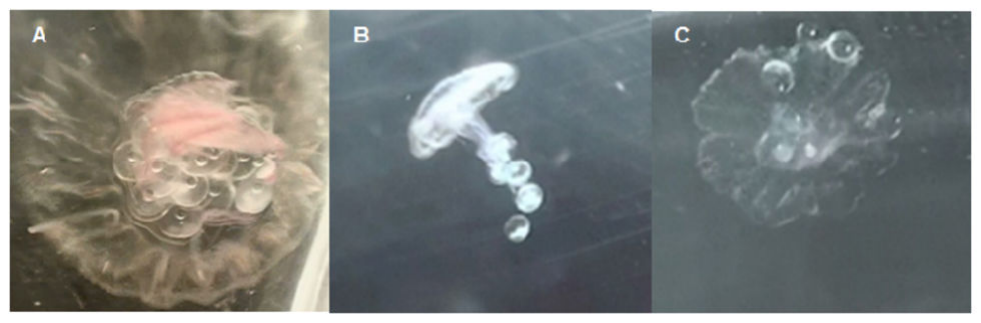
Ephyrae with eggs in different situations: A) ephyra on a Petri disc after feeding, B) ephyra capturing eggs during feeding incubation under stirred conditions, C) ephyra capturing eggs during feeding incubation under unstirred conditions.

IR was significantly affected by the average size of the feeding ephyrae (P<0.001) and by a significant interaction between egg concentration and mixing regime (P=0.026). At low egg concentrations, ephyrae in unstirred beakers had almost twice as many eggs in their guts as ephyrae in stirred beakers (Figure 3). Thus, the ephyrae were particularly apt at removing the eggs from the surface, where they accumulated in the absence of stirring, as is clearly shown in video recordings made during the experiments (Movie S1). However, this difference disappeared at the high food concentrations, resulting in similar IR in both the stirred and the unstirred beakers. This is probably due to the saturation of the digestive system of ephyrae (Figure 4A). Video observations revealed differences in predation mechanisms between mixing regimes: the ephyrae used the subumbellar tentacles when the eggs where homogeneously distributed in the water column and the marginal lappets when the eggs were at the surface (Figure 4B-C).

Ephyrae cleared eggs at rates of up to 6.75 L individual^-1^ h^-1^. The CR was significantly affected by the average size of the feeding ephyrae (P<0.001) and by the interaction between egg concentration and mixing regime (P=0.039, Table 2). The CR of ephyrae in the unstirred beakers was more than twice that of ephyrae in stirred beakers at the low egg concentration (Figure 3). Thus, the larvae were more efficient at removing the 36 eggs used per incubation when accumulated in 60 cm^2^ of water surface than when suspended in the 6 L of water volume. However, this difference disappeared when egg concentration was high, again suggesting some sort of limitation, probably due to gut saturation.

**Figure 5:**
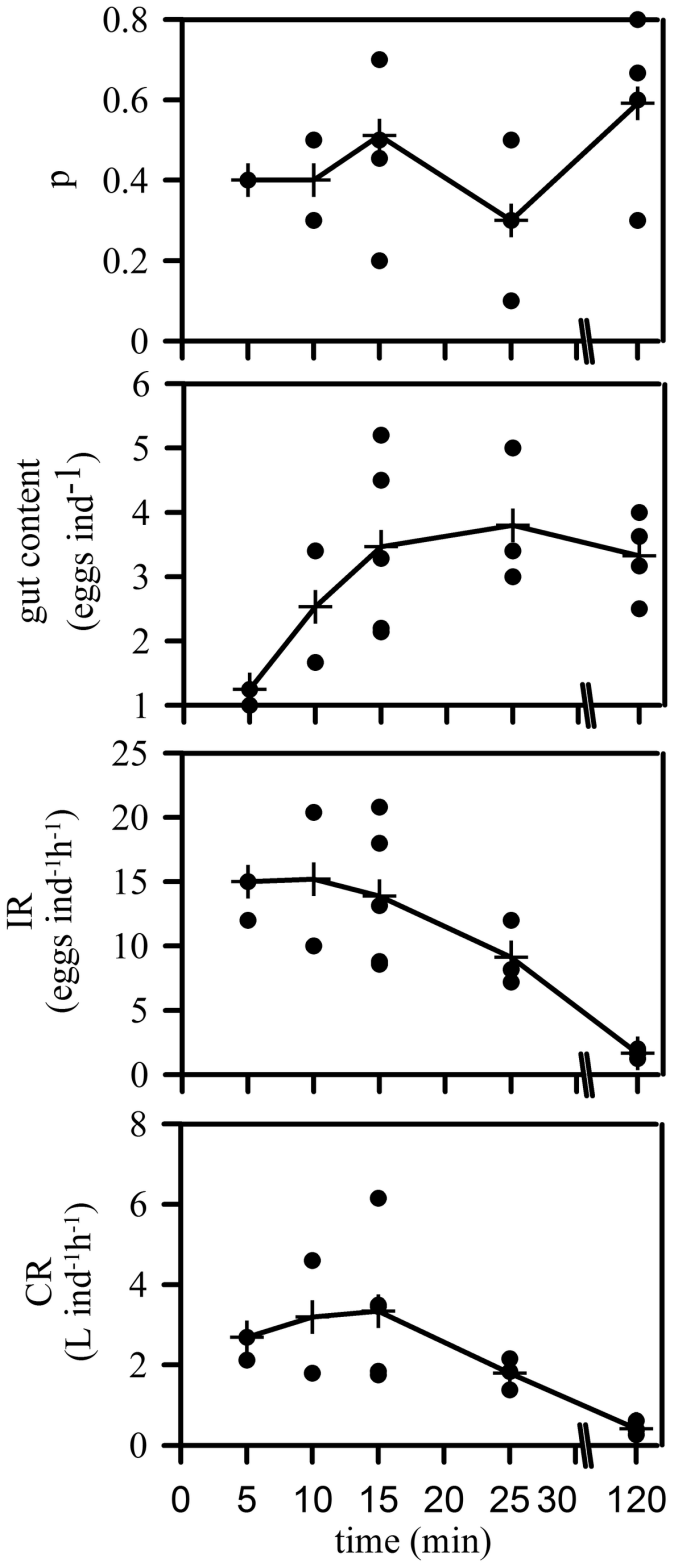
Effect of incubation time on the proportion of feeding ephyrae (p), the number of eggs inside the gastric pouches after incubation, the ingestion rate (IR) and the Clearance Rate (CR). Dots are observations (beakers), and crosses represent means for each incubation time. Crosses have been joined by lines to visualize tendencies.

Although our ANCOVA analysis may to some extent be hindered by differences between treatments in the size range of the ephyrae (see methods), it is fully consistent with the results of the experiment testing the effect of incubation time. The proportion of feeding ephyrae was quite constant for the different incubation times (Figure 5). The number of eggs inside the guts of the ephyrae increased during the first 15 minutes of incubation and showed symptoms of saturation afterwards, at a gut content of nearly 3 eggs ind^-1^ (Figure 5). It should be noted that the conditions during this experiment corresponded to the low egg density/no stirring treatment used in the previous experiment, where we already found symptoms of saturation with an ingestion rate close to 20 eggs ind^-1^ h^-1^ (Figure 3). This ingestion rate is equivalent to a gut content of ca. 5 eggs ind^-1^ after an incubation time of 15 minutes. The ingestion and clearance rates declined after 15 minutes of incubation (Figure 5), basically because the number of eggs inside the guts remained nearly constant, while the incubation time increased.

**Figure 6:**
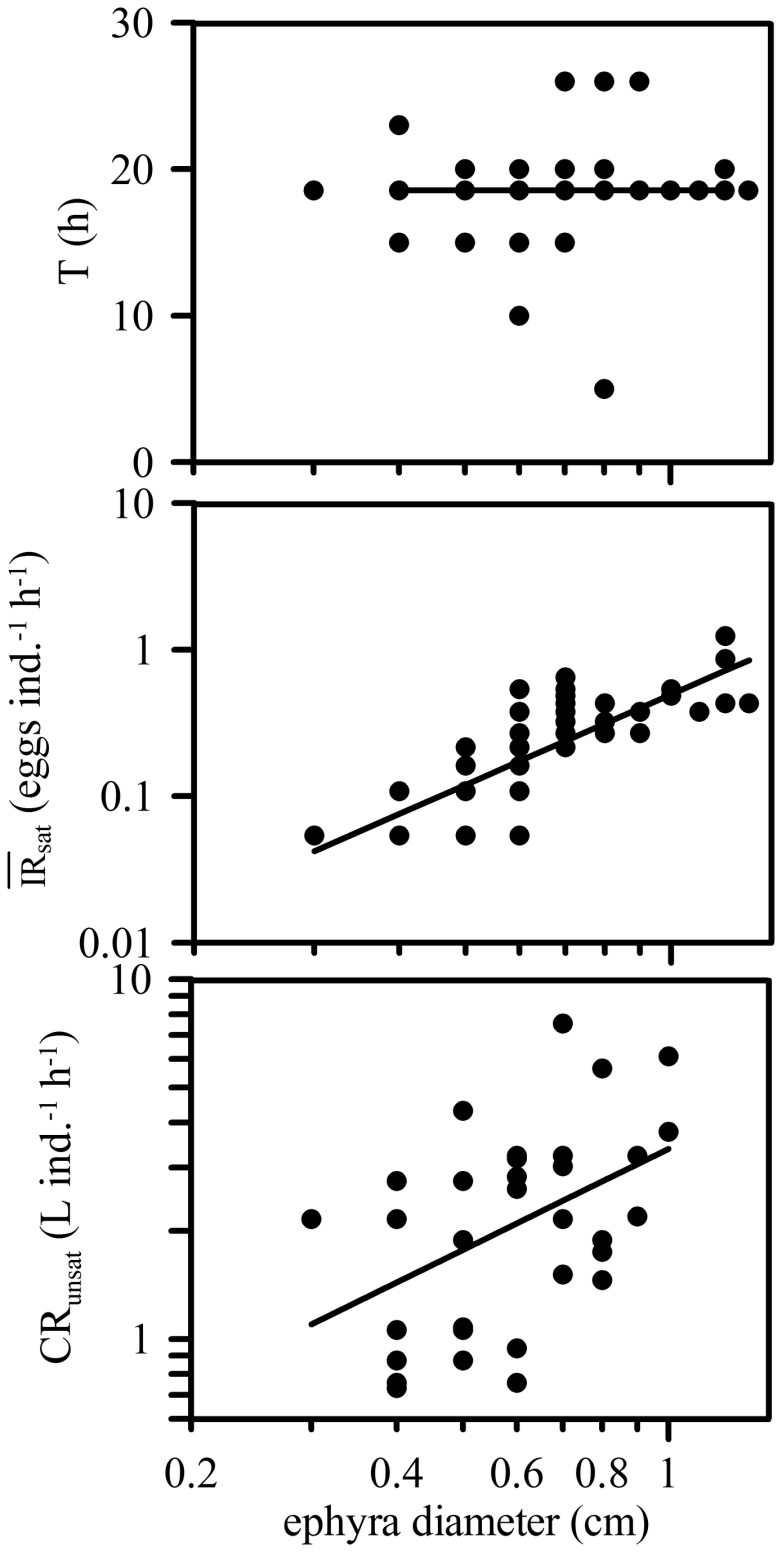
Digestion time (T), average long-term ingestion rate under saturating conditions (${\bar{IR}}_{sat}$) and clearance rate under unsaturating conditions (CR_uns_) vs ephyra length. The solid line indicates: in the upper panel, the mean T (18.55±5.26 h); in the middle panel, the least-squares power regression (log (${\bar{IR}}_{sat}$, eggs ind^-1^ h^-1^)=(-0.305) +(2.05) log (ephyra length, mm), n=70, R^2^=0.65, P<0.001); in the lower panel, the least-squares power regression (log (CR_uns_, L ind^-1^ h^-1^)=(0.529) +(0.934) log (ephyra length, mm), n=35, R^2^=0.176, P=0.006). Animals used for calculation of the ${\bar{IR}}_{sat}$ and the CR_uns_ belong to experimental treatments labeled "(s)" and "(u)" in Table 1.

The digestion time was not significantly related to the size of the ephyra (log-log regression of digestion time on ephyra length, P > 0.05, Figure 6). Therefore, an average digestion time of 18.55±5.26 h can be assumed for all ephyra sizes within the studied size range (Figure 6). This is much longer than the incubation times of our experiments and implies that once full of eggs, the ephyrae must stop ingesting at least until there is sufficient room again for a new egg or, more conservatively, until all the gut contents have been digested. In this scenario, and as a first-order approximation, it follows that the predation rates of *P* *. noctiluca* ephyrae on ABFT eggs should be encapsulated between two extremes: saturating and non-saturating conditions. Under saturating conditions, it is the egg volume and the digestion time that limits the ingestion rate. Under non-saturating conditions, it is the clearance potential and the prey concentration that matters. We will make an attempt at calculating the ingestion rate in saturating conditions and the clearance rate in unsaturated conditions. For this purpose, each animal and its gut content is considered separately, rather than in batches as for the preceding calculations.

An average long-term ingestion rate in saturating conditions (${\bar{IR}}_{sat}$, eggs ind^-1^ h^-1^) can be calculated by assuming that complete digestion is necessary to resume predation. Under these conditions:

$${\bar{IR}}_{sat}=\frac{e_{sat}}{T}$$

where *e*_*sat*_ is the number of eggs inside the full gut of an individual and *T* is the digestion time. ${\bar{IR}}_{sat}$increased rapidly with ephyra length, according to the power relationship; ${\bar{IR}}_{sat}$(eggs in^-1^ h^-1^) = 0.5[ephyra length (mm)]^2^.^05^ (Figure 6).

In unsaturated conditions, the animals would be clearing prey with maximum rates, which can be easily calculated from the number of eggs inside their guts and the egg concentrations in the beakers at the beginning and end of the incubations. Firstly, an instantaneous individual ingestion rate in unsaturated conditions (*IRuns*, eggs h^-1^ individual^-1^) can be calculated as:

$$IRuns=\frac{e_{uns}}{t}$$

where *e*_*uns*_ is the number of eggs inside the ephyra after incubation under non-saturating conditions and *t* is incubation time. Methods in Møller et al. (2010) [47] can then be adapted to estimate an individual clearance rate (L h^-1^ individual^-1^) as:

$$CR_{uns}=\frac{IR_{uns}}{C_{m}}$$

where *C*_*m*_ is the mean egg concentration experienced by the ephyrae inside the experimental container during the course of the incubation [47]. *C*_*m*_ can be estimated as:

$$C_{m}=e^{\frac{\ln\left(C_{0}C_{f}\right)}{2}}$$

CR_*uns*_ calculated using these equations grew almost isometrically with ephyra length, according to the power law CR_uns_ (L ind^-1^ h^-1^) = 3.4[ephyra length (mm)]^0.93^ (Figure 6).

## Discussion

The results of this study clearly demonstrate the predatory capacity of *P* *. noctiluca* on ABFT eggs. Overall, we found four specific experimental results to be discussed in the context of the coupled spatio-temporal distribution patterns of both species in the study area. Firstly, in the lab, large ephyrae are voracious predators of ABFT tuna eggs, with remarkably high clearance rates. Secondly, those clearance rates are sustained or even increased when the eggs are accumulated on the surface of the incubation containers rather than suspended in the water. Thirdly, the ingestion rate of *P* *. noctiluca* ephyrae rapidly saturates, leading to cessation of their prey capture behavior. Finally, *P* *. noctiluca* ephyrae are likely to resume predation after a long digestion time of approximately 18 h.

To our knowledge, *P* *. noctiluca* exhibited among the highest clearance rates reported for jellyfish of a similar size (Table 3). This difference may be in part methodological, as most previous studies measured predation rates by counting the number of prey inside the experimental container before and after incubation (but see 49). However, non-feeding individuals are not detected by this approach, thereby underestimating actual individual clearance rates. Thus, in the present study, the gut contents of all individual ephyrae were inspected right after the incubation, to readily detect non-feeding individuals.

**Table 3:** Experimentally measured clearance rates of small scyphomedusae.

Reference	Predator Species	Predator size	Prey	Prey size/life stage	Clearance rate
Hansson & Kiørboe 2006	*Sarsiatubulosa*	*-*	copepods	-	*0.04-0.10 L h^-1^
Tilves et al. 2013	*Pelagianoctiluca*	3.8-4.2 mm	*Mnemiopsisleidy*	1-3 mm	0.4 L h^-1^
		20-25 mm		8-12 mm	62 L h^-1^
Morand et al. 1987	*Pelagianoctiluca*	8 mm	Artemia	nauplii	*0.13 L h^-1^
Riisgård & Madsen	*Aurelia aurita*	5.1-5.9 mm	Artemia	0.9 mm	0.05-0.09 L h^-1^
Bailey & Batty 1984	*Aurelia aurita*	20-22 mm	*Clupeaarengus*	Yolksac	2.2-5.5 L h^-1^
Fancett &Jenkins 1988	*Cyanea* *capillata*	25 mm	*Paracalunus* *indicus*	*-*	0-2.5 L h^-1^
This study	*Pelagianoctiluca*	5 mm	*Thunnus thynnus*	1 mm	0.73-7.54 L h^-1^

Converted from original units L d^- 1^

In this study, the individual ephyrae cleared actively during rapid ca. 15 min bursts, but halted feeding afterwards. Accordingly, an assumption of steady-state ingestion rate has to be rejected for *P* *. noctiluca* at the prey concentrations and conditions of our experiments, a situation which is similar to that observed by Hanson & Kiørboe ( [50]) in the jellyfish *Sarsiatubulosa* . Our study lends support to their idea that jellyfish use their large gastric pouches as a buffering, food-accumulating organ allowing bursts of high food intake when prey is available in short-lived pulses, as is the case of ABFT eggs during spawning events in the field. During the spawning season in 2012, the average weight of the captured ABFT shoals was 30 t, with a maximum of around 120 t [51]. Spawning takes place at night, with the exact timing varying between ABFT groups, but always taking place within a small temporal window (0: 00 to 3: 00 GMT). Spawning events last about ten minutes, during which the shoals stop horizontal displacement and stay at the surface. According to previous calculations [52] based on average fecundities [53], a 30 t shoal should produce around 840 million eggs within a diameter of approximately 40 m (personal observation). As a result, local egg densities of ca. 668 449 eggs m^-2^ can be reached in the natural environment, 263 times higher than the highest density used in this study. Thus, potential predators present at the right time and location will suddenly experience an extraordinarily abundant prey source before it is dispersed by ocean currents. Clearly, high capture efficiencies and large guts should be highly beneficial under these circumstances.

Short, intense predation episodes by the ephyrae on this pulsed food source might be relevant in their life cycle, in particular in the oligotrophic waters of the Balearic archipelago [54]. This could be especially important for a species like *P* *. noctiluca* , which has no benthic stage and therefore depends exclusively on surface-dwelling prey. It is worth noting that the digestion time of the ephyrae is close to the duration of one day. This suggests that ephyrae digestion times and feeding rates are metabolic adaptations to a diel feeding cycle. This supposition is consistent with the pattern of diel vertical migration in *P* *. noctiluca* [35,43], most probably related to feeding [55].

The highly positive buoyancy of ABFT eggs does not represent a strategy of floating on the sea surface and evading predation by jellyfish and other pelagic predators. In particular, *P* *. noctiluca* is a typical cruising predator that screens the water following a sinusoidal search path [56], generating feeding currents around its umbrella and feeding tentacles [57]. A priori, this mechanism seems suited to operating in a 3D, not 2D, prey distribution. However, our experiments show that *P* *. noctiluca* ephyrae can sustain higher apparent clearance rates when ABFT eggs are accumulated at the water surface, allowing for rejection of our initial hypothesis. Nevertheless, our measured feeding rates in a 2D prey distribution might overestimate feeding rates in the field, because water motions generated by the feeding ephyrae may well have altered the uniform distribution of eggs towards the edges of the beakers, enhancing their catchability. Our own visual observations during the incubations do not suggest that this may have been a major source of bias. Video observations suggest that this species switches from cruising tactile predation with capture at the subumbellar tentacles (Movie S2) to some kind of directed prey-detection mechanism with capture at the marginal lappets (Figure 3B-C, Movie S1). Interestingly, adult *P* *. noctiluca* exhibit a different capture system for non-motile prey at the water surface, based on positioning their bells upside down and moving their oral arms towards the surface film [56]. Video observations illustrated ephyrae feeding behavior, exhibiting controlled and directed movements between ephyrae to displace the competitor in the pursuit of a prey (Movie S1).

Theory indicates that predators should have shorter searching times and higher consumption rates in a 3D than in a 2D prey distribution [45]. This does not seem to apply for *P* *. noctiluca* ephyrae, which seem to be capable of easily switching from a 3D to a 2D configuration. Neustonic 2D predation may increase predation success on an egg population that has already been dispersed but that floats at the surface in the absence of wind. On more general grounds, the *P* *. noctiluca* -ABFT egg system might represent an example of a general mechanism linking pelagic and neustonic food webs. In the absence of wind forcing, planktonic organisms that swim upwards or that have positive buoyancy will accumulate on the surface, thus becoming a valuable food source for both obligate (e.g. *Physalia* *Physalis* , *Velellavelella* or *Porpitaporpita* ) and facultative neustonic predators (e.g. *P* *. noctiluca* ). The onset of wind-generated turbulence on the sea surface will bring many of those organisms back into suspension, where pelagic predators will be at an advantage.

We conclude that in the Western Mediterranean, *P* *. noctiluca* ephyrae are functionally well suited to predating on ABFT eggs, a highly-pulsed, spatially-restricted resource that potentially switches from a 3D to a 2D configuration in the absence of wind-generated turbulence. However, the potential impact of *P* *. noctiluca* on ABFT early life stages must be verified against field observations in future studies. Moreover, the possibility that the annual cyclic fluctuations of ABFT [4] could be related to those observed in *P* *. noctiluca* [34,58,59] should also be investigated.

## Supporting Information

Movie S1 A video showing the interaction between two ephyrae of *Pelagianoctiluca* at the time of catching Atlantic Bluefin tuna eggs used as prey under unstirred experimental conditions.
(WMV)

Movie S2 Video shots illustrating: 3D distribution of eggs under mixing condition, ephyra catching eggs with subumbellar tentacles under mixing conditions and ephyra under a stereo-microscope after feeding experiment.
(WMV)
